# Noncontact Electromagnetic Wireless Recognition for Prosthesis Based on Intelligent Metasurface

**DOI:** 10.1002/advs.202105056

**Published:** 2022-05-07

**Authors:** Hai Peng Wang, Yu Xuan Zhou, He Li, Guo Dong Liu, Si Meng Yin, Peng Ju Li, Shu Yue Dong, Chao Yue Gong, Shi Yu Wang, Yun Bo Li, Tie Jun Cui

**Affiliations:** ^1^ State Key Laboratory of Millimeter Waves Southeast University Nanjing 210096 China; ^2^ Research Center of Applied Electromagnetics Nanjing University of Information Science and Technology Nanjing 210044 China; ^3^ Department of Biomedical Engineering School of Biomedical Engineering and Informatics Nanjing Medical University Nanjing 211166 China

**Keywords:** dynamic electromagnetic focus, gesture recognition, machine learning, programmable metasurfaces

## Abstract

With the development of artificial intelligence and Internet of Things, hand gesture recognition techniques have attracted great attention owing to their excellent applications in developing human‐machine interaction (HMI). Here, the authors propose a non‐contact hand gesture recognition method based on intelligent metasurface. Owing to the advantage of dynamically controlling the electromagnetic (EM) focusing in the wavefront engineering, a transmissive programmable metasurface is presented to illuminate the forearm with more focusing spots and obtain comprehensive echo data, which can be processed under the machine learning technology to reach the non‐contact gesture recognition with high accuracy. Compared with the traditional passive antennas, unique variations of echo coefficients resulted from near fields perturbed by finger and wrist agonist muscles can be aquired through the programmable metasurface by switching the positions of EM focusing. The authors realize the gesture recognition using support vector machine algorithm based on five individual focusing spots data and all‐five‐spot data. The influences of the focusing spots on the gesture recognition are analyzed through linear discriminant analysis algorithm and Fisher score. Experimental verifications prove that the proposed metasurface‐based non‐contact wireless design can realize the classification of hand gesture recognition with higher accuracy than traditional passive antennas, and give an HMI solution.

## Graphical abstract

A non‐contact hand gesture recognition method based on programmable transmissive metasurface is proposed to achieve higher accuracy of classification than traditional antennas, and can be applied for rehabilitation of hand‐amputated patients in the future. Gesture recognition using support vector machine algorithm based on five individual focusing spot data and all‐five‐spot data are carried out. The average classification accuracy is 99.89%.

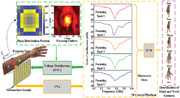

## 1 Introduction

With the development of artificial intelligence (AI) and internet of things (IoT), hand gesture recognition techniques have attracted great deal of attention owing to their excellent application in developing human‐machine interaction (HMI), such as virtual reality control,^[^ ^1^, ^2^ ^]^ prosthesis control^[^ ^3^, ^4^, ^5^, ^6^ ^]^ and rehabilitation.^[^ ^7^ ^]^ Therefore, human gestures can be identified explicitly as input and processed representations through mapping of commands as output for the device control.

Traditional hand gesture recognition systems are generally aided through two types of methods namely vision‐based and contact‐based systems.^[^ ^8^ ^]^ The vision‐based systems mainly adopt vision cameras and depth cameras (such as SoftKinetic HD camera) for data collection.^[^ ^9^, ^10^, ^11^ ^]^ Optical images can describe information such as gesture texture, contour, and shape, but they suffer from configuration complexity and occlusion problems.^[^ ^8^ ^]^ Due to noninvasive and low‐cost merits, surface electromyographic (sEMG) sensors are widely researched and applied for the contact‐based hand gesture recognition systems.^[^ ^3^, ^4^, ^5^, ^6^, ^12^, ^13^ ^]^ sEMG‐based recognition systems can measure the neuromuscular activities from the electrodes placed on the skin of arm, and perform classification algorithms with the time and frequency domain features extracted from the detected sEMG signals.^[^ ^12^ ^]^ In addition, in order to achieve the classification of high accuracy, high‐density sEMG (HD‐sEMG) signals are detected from a high count and high‐density electrode placement, which can have a minimal interference between movements of other fingers and comprehensive spatial coverage.^[^ ^14^, ^15^, ^16^ ^]^ However, contact‐based systems are easily influenced by the signal variations resulting from muscle fatigue and electrode shift.^[^ ^17^, ^18^ ^]^ Moreover, wearable usage is necessary that can reduce the experience and perception of users.

Owing to the characteristics of noncontact, certain penetration, shielding, transmission ability, offering better privacy, and full‐time working conditions of electromagnetic (EM) waves, short‐range noncontact microwave radars^[^ ^19^ ^]^ offer opportunities to realize indoor positioning,^[^ ^20^ ^]^ vital sign monitoring,^[^ ^21^, ^22^, ^23^ ^]^ spatial tracking,^[^ ^23^ ^]^ and fall detection.^[^ ^24^ ^]^ Since Google Inc. designed a 60 GHz millimeter‐wave radar chip to realize multiple gesture recognition,^[^ ^25^ ^]^ the research of hand gesture recognition based on the frequency‐modulated continuous‐wave (FMCW) radar approach has attracted widespread attention.^[^ ^26^, ^27^, ^28^ ^]^ According to the gesture movement distance and velocity response obtained from Doppler radar sensors and the deep convolutional neural network (DCNN), the overall accuracies of more than 90% were acquired for 10 and 14 different hand gestures.^[^ ^27^, ^28^ ^]^ However, the FMCW radar sensor requires using EM waves to directly illuminate the user's hand, and it has not been studied for applying to the HMI of amputated and paralyzed patients.

In refs.[29, 30], Volakis et al. proposed a single on‐body antenna as a sensor for monitoring both the heartbeat and respiration rates. The main idea is to extract the cardiopulmonary information induced on the reflection coefficient of a single antenna placed in proximity of the human thorax.^[^ ^30^ ^]^ Then according to the similar principles, conformal antenna can be used as a sensor to wear on the skin or clothing surface of the amputee, and the movement can be identified by using the slight change in the antenna input impedance during exercise.^[^ ^31^, ^32^ ^]^ However, the muscles that govern finger and wrist movements can cover the entire forearm. The on‐body antenna only has a fixed radiation direction and cannot focus on spatial EM waves. Thus, the distributed muscle activities can not be perceived. In addition, these approaches compromise the user comfort owing to the contacting skin.

Therefore, we propose a novel concept of noncontact EM wireless recognition system based on a transmissive intelligent metasurface for a multifunctional upper‐limb prosthesis. The metasurfaces are defined as subwavelength 2D periodic unit structures at surfaces or interfaces with different geometries and distributed functional arrangements.^[^ ^33^, ^34^, ^35^ ^]^ Since digital coding and programmable metasurfaces^[^ ^36^ ^]^ were introduced to dynamically manipulate different scattered EM wave modulations, a series of information metasurface systems have been constructed with novel applications and large potentials,^[^ ^37^ ^]^ such as holograms,^[^ ^38^ ^]^ imaging,^[^ ^39^ ^]^ and new architecture wireless communication systems.^[^ ^40^, ^41^, ^42^, ^43^ ^]^ Recently, an intelligent prototype system based on the reflective metasurface and DCNN technology was proposed to realize in situ full‐scene imaging and adaptive recognition of hand signs and vital signs of multiple noncooperative people.^[^ ^39^ ^]^ However, compared with C‐band, the lower working frequencies (such as 2.4 GHz) in the L‐band will result in the larger unit and array sizes, which are insufficient to detect the slight movement of forearm muscles. More importantly, the feed blockage of the reflective metasurface is not convenient for testing and will limit its further application.

**Figure 1:**
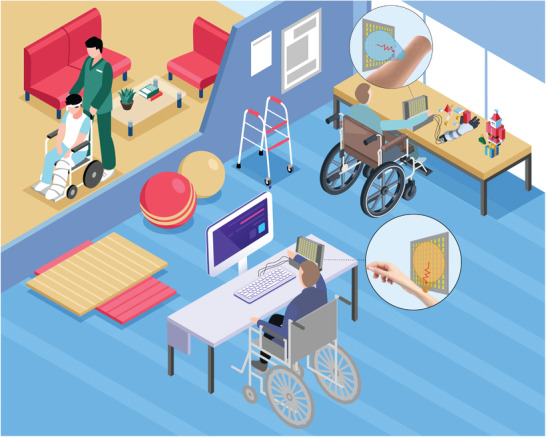
The future application illustrative scenario for recognizing gestures of hand and wrist in the rehabilitation of patients with hand amputation and upper limb paralysis. The intelligent metasurface‐based non‐contact system can be used as a new option for the human–machine interaction (HMI). By taking advantage of its EM wave focusing ability in the wave front engineering, the programmable metasurface is employed to directly illuminate the forearm. The RF signal can be emitted into the investigated region through the transmisson‐type metasurface, and then the echoes bounced back from the human are detected through the same metasurface. The varied echo coefficients obtained from the intelligent metasurface is used for the gesture recognition of hand and wrist, empowered by using machine‐learning algorithm. Thus the patients can use this intelligent metasurface system to control the prosthetic hand or finish the rehabilitation training in real‐time.

Here, we propose a noncontact hand gesture recognition method by using a transmissive programmable metasurface working in C‐band to directly illuminate the forearm of user. The EM signal can be emitted into the investigated region through the transmission metasurface, and then the echoes bounced back from the human can be detected through the same metasurface. The variations of near field perturbed by finger and wrist agonist muscles can be mapped in the corresponding echo coefficients, which can be used to identify hand movements patterns. So the intelligent metasurface is deployed to directly illuminate the forearm instead of hand, with taking advantage of its EM wave focusing ability in the wavefront engineering. The programmable metasurface can be dynamically controlled to converge EM waves to much more spots according to the anatomical muscle distribution. Therefore, compared with traditional antennas, the echo coefficients variations acquired from programmable metasurfaces contain much more muscle movement information that may achieve the gesture recognition of higher accuracy. A future application illustrative scenario for recognizing gestures of hand and wrist in the rehabilitation of patients with hand amputation and upper limb paralysis is presented in **Figure** **1**. This intelligent metasurface introduces a new noncontact and electromagnetic wireless strategy to recognize the gestures of upper limb prostheses without any contact‐based electrodes or sensors. In this sense, our method can be used as a new option for intelligent human–machine interaction.

## 2 Results and Discussion

### 2.1 Working Principle of the Intelligent Metasurface‐Based Gesture Recognition

**Figure 2:**
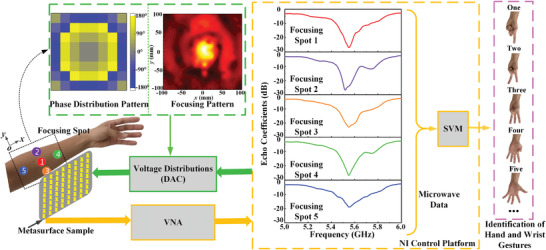
The working principle of the non‐contact hand gesture recognition method based on the programmable metasurface sample with a machine learning algorithm. The five different voltage distributions corresponding with phase distribution patterns can be obtained for controlling the transmissive programmable metasurface so that its radiation wave is focused onto the desired spots.

The concept of a noncontact hand gesture recognition method based on the programmable metasurface sample with a machine learning algorithm is illustrated in **Figure** **2**. The utilized transmission programmable metasurface sample is composed of 8 × 8 meta‐atoms (≈3.83*λ* × 3.83*λ*). Each meta‐atom has a size of 25 mm × 25 mm, with integration four varactor diodes (MA46H120)^[^ ^44^ ^]^ for the control of the transmission phase. Details on the designed meta‐atoms of the programmable metasurface are provided in S1 (Supporting Information). The metasurface focusing is achieved by compensating the phase difference between unit cells, so that the units reach a focal point in the near‐field region with the same electronic distance. Therefore, in order to generate the focusing points, the *mn‐th* unit phase compensation *Φ*_mn_ of the metasurface at the focal point (*x*_0_, *y*_0_) can be written as:

(1) $$\Phi_{mn}\left(x_{0},y_{0}\right)=k_{0}\left[\sqrt{F^{2}+{\left(x_{m}-x_{0}\right)}^{2}+{\left(y_{n}-y_{0}\right)}^{2}}-F\right]+\Phi_{0}$$

where *k*_0_ is wave number, and *F* is the focal length. (*x*_0_, *y*_0_) represents the focus position on the *x‐* and *y‐*axis, and (*x_m_*, *y_n_*) represents the position of the unit in the *m‐th* row and *n‐th* column on the *x‐* and *y‐*axis. To generate the focal points in the near‐field, the phase distributions of the proposed metasurface can be obtained by compensating the space distance between the unit cells. Here, the focal length is 100 mm, corresponding to the distance from the programmable transmission metasurface and the forearm targets. In addition, the transmission phase of the designed meta‐atom has good linearity with the capacitance of the varactor. Therefore, the voltage distribution mapping with the phase distribution can be determined for controlling the transmissive programmable metasurface, and the radiation EM wave from the metasurface can be dynamically focused onto the desired spot. In addition, the feed source of a patch array antenna with a power divider network is employed to transmit the excitation of quasi‐plane wave to each meta‐atom, and the RF SMA is connected with one port of the vector network analyzer (VNA). The microwave raw data (echo coefficients) can be obtained by measuring the S_11_ parameter through the VNA when the radiation waves are controlled to focus on different spots. Finally, the support vector machine (SVM) algorithm is introduced to process the microwave raw data to finish the gestures recognition of hand and wrist.

### 2.2 Dataset Setup

**Figure 3:**
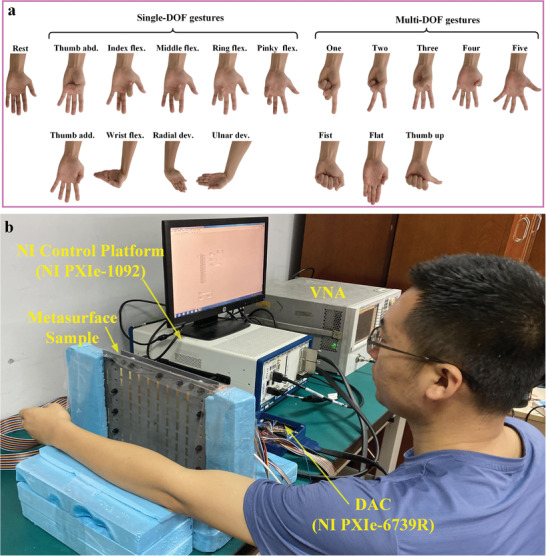
Dataset and experimental setup. a) The 18 gestures of hand and wrist employed in this study include single‐DOF gestures and multi‐DOF gestures. The single‐DOF gestures subset includes individual finger flexions, wrist flexions and deviations along with the “rest” gesture. The single‐DOF gestures are “thumb abduction (abd.),” “index flex.,” “middle flex.,” “ring flex.,” “pinky flex.,” “thumb adduction (add.),” “wrist flex.,” “radial dev.” and “ulnar dev.” The multi‐DOF gesture subset includes typical‐use, isometric hand gestures including multiple finger abduction and adduction. The multi‐DOF gesture are “one,” “two,” “three,” “four,” “five,” “fist,” “flat” and “thumb up.” b) Photograph of the experimental setup for the intelligent metasurface‐based gesture recognition.

**Figure 4:**
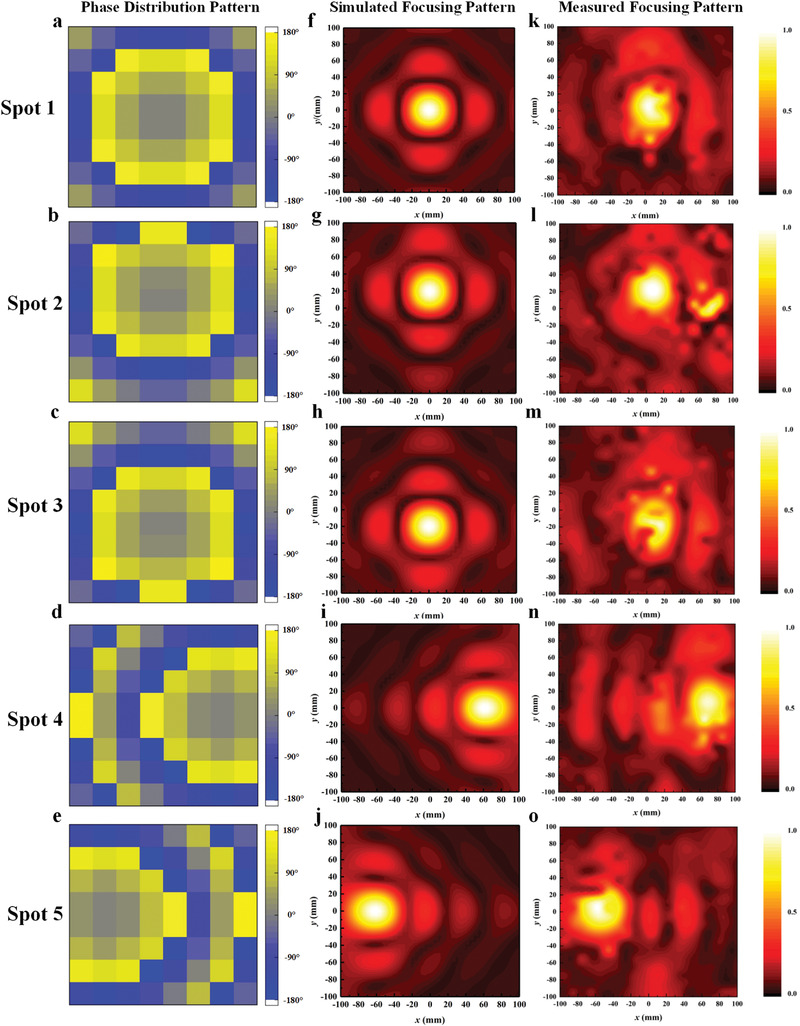
Phase distribution patterns, corresponding simulated and measured near‐field focusing patterns of the metasurface sample for focusing the radiation EM wave onto the five desired spots.

**Figure 5:**
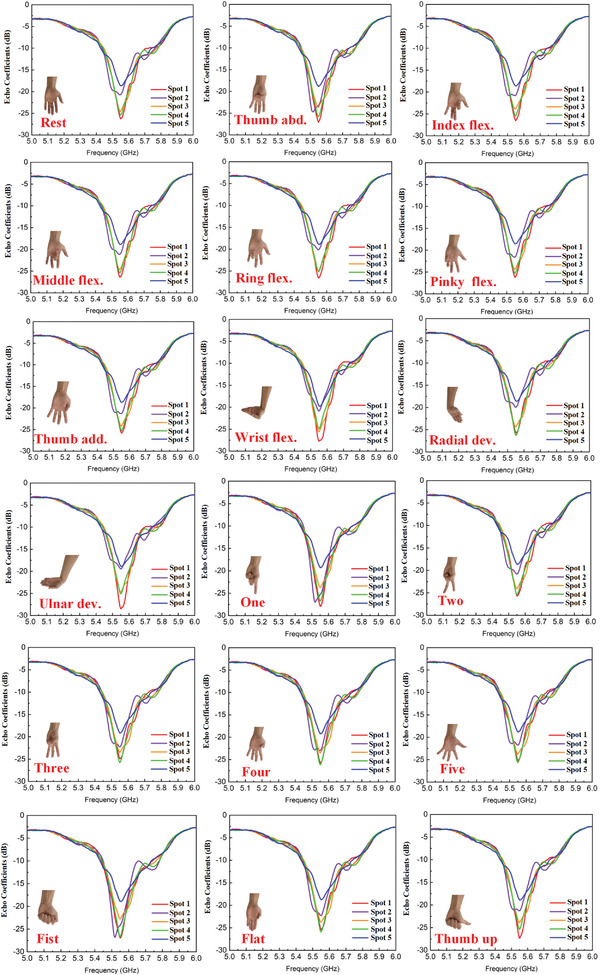
Examples of the microwave data obtained for 18 gestures in the dataset. In each subfigure, it illustrates five echo coefficients curves, where 201 frequency points from 5 to 6 GHz are utilized for each spot with the same gesture.

The experimental setup for the intelligent metasurface‐based gesture recognition is provided in **Figure** **3**. Please refer to Experimental Section for more information about the experimental setup. Using the setup prototype, we collected and analyzed an offline microwave dataset from one healthy participant across multiple gestures of wrist and hand. As illustrated in Figure 3a, 18 gestures of hand and wrist employed in this study include single‐degree of freedom (DOF) gestures and multi‐DOF gestures. The single‐DOF gestures subset includes individual finger flexions (flex.), wrist flexions and deviations (dev.) along with the “rest” gesture. The multi‐DOF gesture subset includes typical‐use, isometric hand gestures including multiple finger abduction and adduction. Considering the anatomical muscle distribution of the forearm, five different focusing spots are selected in this experiment. As shown in Figure 2, the inner forearm is mapped to a 2D plane, where *x* and *y* axes are parallel and perpendicular to the direction of the arm, respectively. The Spot 1 is defined as a reference point, which is directly corresponding to the center of the metasurface sample. **Figure** **4**a–e shows the phase distribution patterns of the five spots. According to Huygens’ principle, each unit cell can be considered as an ideal source in the simulation. Therefore, by calculating Equation (1) to obtain the phase distribution of the transmission metasurface for near‐field focusing, the electromagnetic energy distribution on the focal plane can be analyzed by solving the scalar Green's function in free space. The energy distributions of the five focal points are simulated in MATLAB 2018.0 with the setting distance from the metasurface to the focusing plane as 100 mm, and the simulated results are shown in Figure 4f–j. By using digital–analog conversion (DAC) modules, the patterns of phase distribution can be converted to the corresponding biasing voltage of varactors, and then the metasurface can dynamically change the focusing spot of EM wave in real time. The metasurface sample is measured by a 2D near‐field test scanning platform, and the focusing patterns of five spots are shown in Figure 4k–o. It can be observed clearly that the locations of the five spots are (0, 0), (0, 20), (0, ‐20), (60, 0), and (‐60, 0) with the unit of (mm, mm). For each gesture, the subject was required to keep stable when continuously recording 10 microwave data samples under every focusing spot illuminated by the transmissive programmable metasurface. Each gesture was measured 15 times. Therefore, we obtained 2700 samples in total for the 18 gestures. Examples of the microwave data obtained are presented in **Figure** **5**. In each subfigure, it illustrates five echo coefficients curves, where 201 frequency points ranging from 5 to 6 GHz are utilized for each spot with the same gesture.

### 2.3 Experimental Verification Method

We suppose a better accuracy of gesture recognition can be achieved using our metasurface‐based strategy than antennas with fixed‐radiation direction, owing to the controllable EM focusing ability that can provide multiple muscle information. To investigate our hypothesis, three steps are proposed for the verification: 1) By using the one‐versus‐one SVM (OVO‐SVM) algorithm, the gesture recognition success rate is based on the all‐five‐spot data and the five individual focusing spot data are tested. Through this step, we can preliminarily investigate the influence of each focusing spot to the gesture recognition accuracy. More details on the SVM algorithm flow including the training phase and testing phase are provided in S2 (Supporting Information) . 2) The linear discriminant analysis (LDA) algorithm is used to reduce the dimensionality of the dataset and a qualitative analysis is performed to inspect the class feature distribution of each focus spot. 3) Fisher score is employed to quantitatively analyze the feature contribution of each focusing spot, so as to discover the influence of the focusing spot on the accuracy of gesture recognition. Fisher score *J*(*W*) is defined as follows:

(2) $$J\left(W\right)=\mathrm{Tr}\left\{{s_{W}}^{-1}s_{B}\right\}$$

(3) $$s_{W}=\sum_{k=1}^{K}\sum_{n\in C_{k}}\left(y_{n}-\mu_{k}\right){\left(y_{n}-\mu_{k}\right)}^{T}$$

(4) $$s_{B}=\sum_{k=1}^{K}N_{k}\left(\mu_{k}-\mu \right){\left(\mu_{k}-\mu \right)}^{T}$$

where *S*_W_ and *S*_B_ are within‐class scatter matrix and between‐class scatter matrix, respectively. The *C_k_* is the index set of the *k*‐_th_ class samples, *N_k_* is the number of *k*‐_th_ class samples, and there is a total of *K* classes. The *y_n_* is the *k*‐_th_ class sample set, *μ_k_* is the mean value of the *k*‐_th_ sample, and *μ* is the mean of all samples. In short, higher Fisher score indicates that certain feature has higher between‐class dispersion and lower within‐class dispersion, thereby, it contributes more to the separability. Fisher scores for all types of movements under the five focusing spots are calculated. In addition, because the wrist flexors and finger flexors are distributed in different areas of the forearm, Fisher scores for wrist‐type gestures and finger‐type gestures are also provided to determine which focusing spot has a better feature separability contribution for a certain type of gestures (all‐type, wrist‐type, finger‐type). More details on the LDA algorithm and feature extraction algorithm used for the Fisher score are provided in S3 and S4 (Supporting Information).

In fact, the active muscles are divided into deep muscles and superficial muscles, and there are still some differences for individuals. Therefore, the anatomical structure of the wrist and hand movement agonists can be used for preliminary localization. In the initial learning stage, multichannel small sample size data collection is performed by setting multiple focal points, and the channel feature evaluation is performed using quantitative indicators of class separability such as Fisher score, Bhattacharyya distance, Shannon entropy, etc. Then the top 50% focusing points with class separability contribution are selected. Finally, we then hope to reduce the overall number of training samples and training time while maintaining recognition accuracy with fewer channels of sample collection. In order to verify its feasibility, we selected three focusing spots according to the value of the Fisher score. Then average classification accuracies between single‐spot, triple‐spot and all‐five approaches are compared statistically. Please refer to Experimental Section for more information about statistics.

### 2.4 Gesture Recognition Results Based on SVM Algorithm

**Figure 6:**
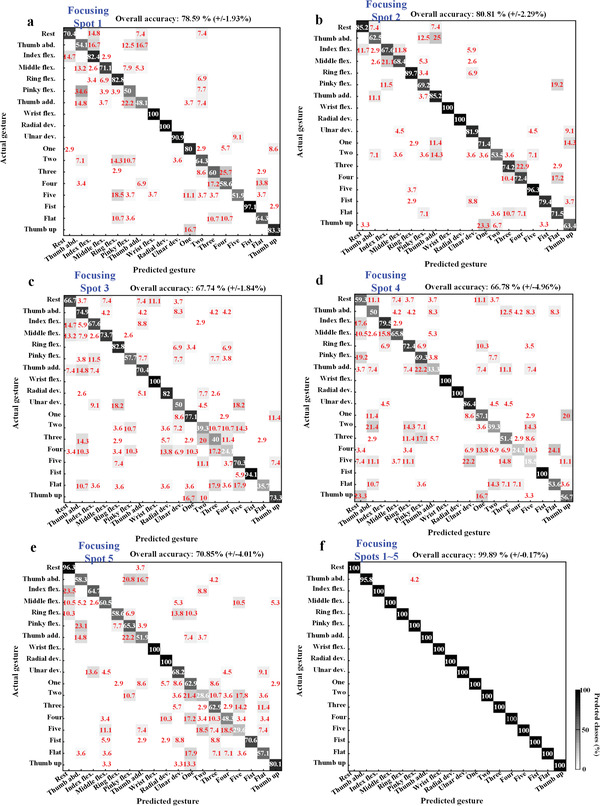
Classification confusion matrices of gesture recognition test based on SVM algorithm under different focusing spot data. The white number values in the matrix represent percentages of correct predictions, while the red values are percentages of incorrect predictions. The gray‐scale color‐bar and background represent the performance and the proportion of predicted classes. a–e) Classification test results based on Spot 1, Spot 2, Spot 3, Spot 4, Spot 5 data. f) Classification test results based on all‐five‐spot data.

The experimental verification of gesture recognition based on OVO‐SVM algorithm is performed using the dataset containing 2700 recorded samples for the 18 gestures. This dataset is divided into 80% for the SVM training and 20% for the testing with ten‐fold validation. A python program is used to execute the experimental verification. We take the result of subject A as an example to illustrate. The classification confusion matrices of gesture recognition test based on SVM algorithm under different focusing spot data are illustrated in **Figure** **6**. The average overall classification accuracies under individual focusing spot data are as follows: 78.59% ± 1.93% (Spot 1), 80.81% ± 2.29% (Spot 2), 67.74% ± 1.84% (Spot 3), 66.78% ± 4.96% (Spot 4), 70.85% ± 4.01% (Spot 5). Simultaneously, it can be observed that the gesture recognition under all‐five‐spot data has the best performance, and it only mispredicts “thumb abduction” as “pinky flexion.” Thus, the average overall classification accuracy under tenfold validation is 99.89% ± 0.17%. According to the confusion matrix of the dataset based on Spot 1 and Spot 2, the recognition success rate of the dataset under these two focusing spots is relatively similar. While the main predicted error in Spot 2 occurs in single‐DOF finger gestures (“thumb abduction,” “index flexion,” “middle flexion,” and “pinky flexion”), and one in Spot 1 is multi‐DOF gestures (“two,” “three,” “four,” “five,” and “flat”) and single‐DOF gestures (“thumb abduction,” “pinky flexion” and “thumb adduction”). Finally, compared with the above two spots, the dataset based on Spot 3, Spot 4, and Spot 5 all have much more errors in the classification of finger and wrist movements. However, among the five individual spots, the Spot 3 has the highest prediction accuracy in the “thumb abduction” and “middle flexion,” the Spot 4 performs best in the “fist” and “pinky flexion”, and the Spot 5 has the highest “rest” classification accuracy. The mean classification accuracies ± SD under the individual subject dataset training with all‐five focusing spots can achieve 99.89 ± 0.17% (Subject A), 99.44 ± 0.48% (Subject B), and 96.52 ± 1.1% (Subject C). Therefore, more muscle information can be obtained through using multiple focusing spots, which can effectively improve the performance of gesture recognition. While after merging three subjects’ data into one database, the classification accuracy decreased to 91.36 ± 0.95% using all‐five focusing spots.

### 2.5 Influence Analysis of the Focusing Spot on the Gesture Recognition

**Figure 7:**
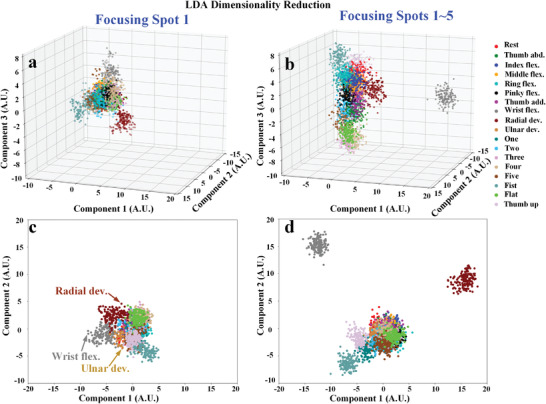
Feature reduction analysis results based on linear discriminant analysis (LDA) algorithm. (a) (b) 3D feature distribution view based on focusing spot 1 and all‐five‐spots. (c) (d) 2D feature distribution view based on focusing spot 1 and all‐five‐spots.

**Figure 8:**
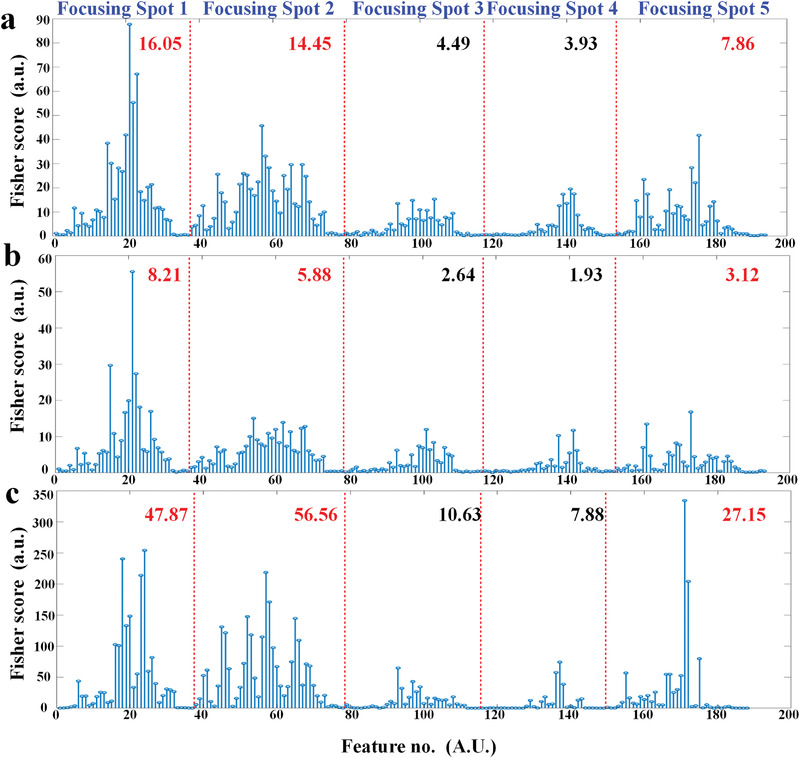
Calculated Fisher score of each extracted feature and the average result of Fisher score for each spot. a) Fisher score calculated based on the dataset of all 18 types of gestures. Fisher score calculated based on the dataset of b) wrist‐type gestures and c) finger‐type gestures.

In order to qualitatively analyze the influence of focusing spot on the gesture recognition, the dimensionality of the microwave data in the dataset is reduced to three and two dimensions by using LDA algorithm. **Figure** **7** shows the 3D and 2D feature distribution views based on dataset under focusing Spot 1 and all five spots. As illustrated in Figure 7a,c, it can be observed that the dataset based on Spot 1 is more distinguishable for wrist‐type movements including wrist flex. and radial dev., but less distinguishable for finger‐type movements. While by using all‐five‐spot data, it can make the distinction of finger‐type movements greatly improved (Figure 7b,d). In addition, calculated Fisher scores of each extracted feature and the average result of the Fisher score for each spot are illustrated in **Figure** **8**. It can be seen that the average result of Fisher score for Spot 1and Spot 2 is the top two scores in the dataset of all 18 types of gestures (Figure 8a). Furthermore, the two spots also occupy the highest two positions in the dataset of wrist‐type gestures (Figure 8b) and finger‐type gestures (Finger 8c). This can explain why the classification success rate of Spot 1 and Spot 2 is higher than others. While the Spot 3, Spot 4, and Spot 5 all have lower average result of Fisher score. It means the contribution of class separability is low, which leads to the worse classification accuracy. According to the above qualitative and quantitative analysis, because of muscles controlling the finger and wrist movements are covering the entire forearm, the microwave data perceived under the individual focusing spot can only make a partial contribution to the divisibility, and cannot provide enough information for classifying finger‐type and wrist‐type movements. Therefore, due to the ability of dynamic controlling the EM focus in the wavefront engineering, the transmissive programmable metasurface is used to illuminate the forearm with more focusing spots and obtain comprehensive data for improving hand and wrist gesture recognition.

**Figure 9:**
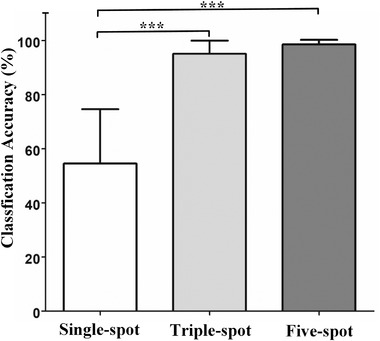
The average classification accuracy comparison between single‐spot, triple‐spot, and all‐five approaches. The results are shown as the mean classification accuracy ± SD (%) (*n* = 30). ∗∗∗*P* < 0.001, as determined by one‐way ANOVA with comparison using post‐hoc test (LSD).

**Figure** **9** shows the average classification accuracy comparison between single‐spot, triple‐spot, and all‐five approaches. In the trial of triple‐spot group, three top‐ranked focus points for Fisher score are selected as the input data to obtain the corresponding recognition accuracy. The mean classification accuracy of triple‐spot group (95.12 ± 4.89%) and five‐spot group (98.61 ± 1.70%) are significantly higher than one for single‐spot group (54.54 ± 20.08%), respectively (∗∗∗*P* < 0.001). While there is no significant difference between the triple‐spot approach and all‐five analysis (*P* = 0.262). The above results show that the specific anatomical knowledge and channel feature evaluation with quantitative indicators of class separability (such as Fisher score) can be used to optimize the number and positions of selected focusing spots. It has a good complexity‐accuracy tradeoff and can maintain recognition accuracy while reducing the number of training samples.

### 2.6 Discussion

As far as the authors know, there is no reference investigating which frequency band is most sensitive for the echo coefficients to the changes of finger and wrist agonist muscles. For the millimeter‐wave band, it has the advantages of better position‐accurate and biologically friendly to be used. However, it is challenging and not easy to design a reconfigurable and programmable metasurface in a millimeter‐wave band due to the small wavelength, small unit size, and difficult active device integration technology. While the dynamic focusing technology based on microwave‐band programmable metasurface is relatively more mature. In addition, in our work, it has the requirement of simultaneously detecting the deep and superficial muscles of the finger and wrist. As tissue absorption increases with frequency, most analyses assume that lower frequency would yield better power transfer efficiency.^[^ ^45^ ^]^ According to the ref. [46], the approximated optimal transfer frequencies for the tissues (such as muscle, skin, and fat) are in the range from 3.93 GHz to 8.64 GHz assuming a distance with 1 cm. They are above 1 GHz even for a distance with 10 cm.^[^ ^46^ ^]^ Furthermore, compared with C‐band, the lower working frequencies (such as 2.4 GHz) in L‐band will result in larger unit and array sizes. Therefore, the C‐band metasurface is selected for this validating design and experiment.

Nevertheless, there are still three aspects to be considered to improve this research:

1) The conventional noncontact methods, e.g., the millimeter‐wave radar measurements, they have been proved that dynamic hand gesture recognition can be achieved through millimeter wave radar and machine learning technique. The above methods need using EM waves to directly illuminate the user's hand, while there is no reference investigating hand gesture recognition through using millimeter wave radar directly to illuminate the forearm. Compared with conventional noncontact methods, our proposed method has the disadvantage that the arm has to be steady and placed very close to the metasurface sample within 10 cm during the test, which is not quite comfortable especially for patients. However, because this is a validating design, and the main purpose is to verify our design principle of a non‐contact hand gesture recognition method based on intelligent metasurface through the prototype and experimentation. Therefore, in the current experiments, the proposed metasurface approach is limited in the static training/data acquisition for hand gesture recognition. Hence the design of conformal metasurface and recognition algorithm for wearable and dynamic movement recognition scenarios need to be further researched.
2) It can be seen that the proposed approach works well for a single‐subject training. The actual system should generalize to any patient with possibly very different arm shapes. Therefore, at present, it is difficult to achieve accurate classification and recognition across individuals, and it can be a customized strategy for a specific patient. We consider that collecting more data from different people, dynamically scanning with more points using a programmable metasurface and designing a deep neural network model can possibly help to address the cross‐individual issue and generalize to more patients in the future study.
3) We believe that using a multifocal point approach^[^ ^47^ ^]^ is likely to help in improving the hand gesture recognition accuracy and reducing the detection time. In the future study, the coding metasurface with multifocal point approach will be considered for investigating recognition performance.

## 3 Conclusion

We propose a noncontact method by using a transmissive programmable metasurface to realize the hand gesture recognition. The echo coefficients under five different focusing spots controlled by the programmable metasurface are recorded and 18 movements of hand and wrist are successfully recognized under the frequencies from 5 to 6 GHz. The success rate of classification based on the all‐five‐spot data and the five individual focusing spot data are tested using OVO‐SVM algorithm, and the influences of the focusing spot on the gesture recognition are qualitatively and quantitatively analyzed through LDA algorithm and Fisher score. The number and positions of selected focusing spots can be optimized through specific anatomical knowledge and channel feature evaluation with Fisher score. It can realize a complexity‐accuracy tradeoff and can maintain recognition accuracy while reducing the number of training samples. Therefore, compared with traditional passive antenna, the unique echo coefficients variations resulting from the near field perturbed by finger and wrist agonist muscles are obtained through programmable metasurfaces by controlling the EM focus. The experimental verifications are implemented to prove that this intelligent metasurface‐based noncontact hand gesture recognition method can contribute to higher accuracy of classification, and give an HMI solution, which can be applied to hand amputation prosthetic arm.

## 4 Experimental Section

The setup for measuring the microwave data is shown in Figure 3b. Three male healthy subjects (26 ± 7.9 years) participated in the experiment. The left arm of the healthy subject (male, 34 years) was placed on the same height position with the center of the transmissive programmable metasurface sample. The distance from the arm to the metasurface sample is 10 cm. In this metasurface system, two high‐speed DAC modules (PXIe‐6739, National Instruments Inc.) are applied to convert phase distribution patterns to the corresponding biasing voltage of varactor diodes on the metasurface units in real time. Assuming varactor diodes in meta‐atoms respond simultaneously, the total time for dynamic switching of five focusing points requires approximately 100 us. SMA located at the feed source of the metasurface sample was connected with one port of VNA (Keysight N5230C). The power level of the vector network analyzer was set as ‐5 dBm. According to the measured focusing pattern (Figure 4), the radius of each focusing spot in the illuminated forearm is about 2 cm. Under the assumption of ideal transmission metasurface, the power density *S* for the focus point can be calculated as *S* = ‐5 dBm / (pi*4 cm^2^) = 0.0247 mW cm^‐2^. While the averaged power density limit for the general population/uncontrolled exposure in 30 min within the frequency range of 1.5 and 10 GHz is 1 mW cm^‐2^, which is illustrated in FCC OET Bulletin.^[^ ^48^ ^]^ Considering that the proposed metasurface has insertion loss (0.9 to 2.3 dB at the frequency of 5.65 GHz in Figure S2, Supporting Information) and all focus points are illuminated and switched in dynamic with a shorter time duration, the power level of the design and experiment meets the national standard of microwave radiation safety. The power level of the VNA was set as ‐5 dBm. The EM echoes data bounced back from the subject can be obtained by measuring the S_11_ parameter through the VNA when the radiation waves are controlled to focus on different spots. A control platform (PXIe‐1092, National Instruments) composed of a PC with LabView 2018 customized‐designed software is utilized to control the DAC modules and record microwave data from the VNA.

### Statistical Analysis

There are three groups, including single‐spot, triple‐spot, and five‐spot group. In each group, the classification accuracies under tenfold validation are calculated for three subjects. Each subject has ten accuracy results. The calculated results are presented as mean ± SD. Therefore, sample size (n) is 30 for the statistical analysis. A one‐way analysis of variance (ANOVA) with comparison using post‐hoc test (LSD) is implemented on classification accuracy result, and the factor is number of used focusing spot. The purpose is to study whether there is a significant difference between three groups. The statistical analysis is performed with SPSS statistics 25 software (IBM corp., Chicago, IL, USA). The results are reported as the mean ± SD. Differences with *P* ≤ 0.05 are considered significant.

## Conflict of Interest

The authors declare no conflict of interest.

## Supporting information

Supporting Information

## Data Availability

The data that support the findings of this study are available from the corresponding author upon reasonable request.
