# Supplementary material for: Noncontact Electromagnetic Wireless Recognition for Prosthesis Based on Intelligent Metasurface
Source: Adv Sci (Weinh). 2022 May 7;9(20):2105056. doi: 10.1002/advs.202105056 (PMC9284131; doi:10.1002/advs.202105056)
Supplement: Supplementary file 1 — Supporting Information [file ADVS-9-2105056-s001.pdf]

ADVANCED SCIENCE
Open Access

Supporting Information

for *Adv. Sci.*, DOI 10.1002/advs.202105056

Noncontact Electromagnetic Wireless Recognition for Prosthesis Based on Intelligent Metasurface

Hai Peng Wang, Yu Xuan Zhou, He Li, Guo Dong Liu, Si Meng Yin, Peng Ju Li, Shu Yue Dong, Chao Yue Gong, Shi Yu Wang, Yun Bo Li and Tie Jun Cui**

Supporting Information

Non-contact electromagnetic wireless recognition for prosthesis based on intelligent metasurface

Hai Peng Wang#, Yu Xuan Zhou#, He Li#, Guo Dong Liu, Si Meng Yin, Peng Ju Li, Shu Yue Dong, Chao Yue Gong, Shi Yu Wang, Yun Bo Li, and Tie Jun Cui**

S1. Design of the meta-atom of the transmission-type programmable metasurface

S2. SVM algorithm processing flow for the gesture recognition

S3. Linear discriminant analysis (LDA) for multiple classes

S4. Feature extraction algorithm used for Fisher score

S1. Design of the meta-atom of the transmission-type programmable metasurface

The geometry of ultilized transmission-type programmable metasurface unit is shown in **Figure S1**. The overall structure is composed of three printed circuit boards (PCB), which are fabricated on the substrate F4B. The middle layer is a multi-layer PCB with the dielectric constant ε_r = 2.65, and dielectric constant of other layers is 2.2. The meta-atom is mainly consists of three parts, including receiving antenna, phase shifter and transmitting antenna (Figure S1a). The size of the receiving antenna and transmitting antenna are absolutely consistent. A rectangular patch antenna (Figure S1d and e) and a bow-tie antenna (Figure S1c) are integrated with the air is inserted between the two anntennas to increase the working bandwidth effectively. By optimizing the size of the antenna structures, the resonant frequency of the antennas can be adjusted the same with the one of the incident wave, which can achieve better performance for the receiving and radiating the electromagnetic (EM) signals. Reflection-type phase shifter is widely used due to its simple structure and excellent matching between the input and output. The basic circuit of the electrically reconfigurable phase shifter is shown in Figure S1d, which contains a directional 3dB coupler and a

reflective terminal load. The four MA46H120 varactor diodes (MACOM Inc.) are integrated in the 3 dB branch line hybrid coupler and a new type of resonant circuit to realize an electrically reconfigurable phase shifter.

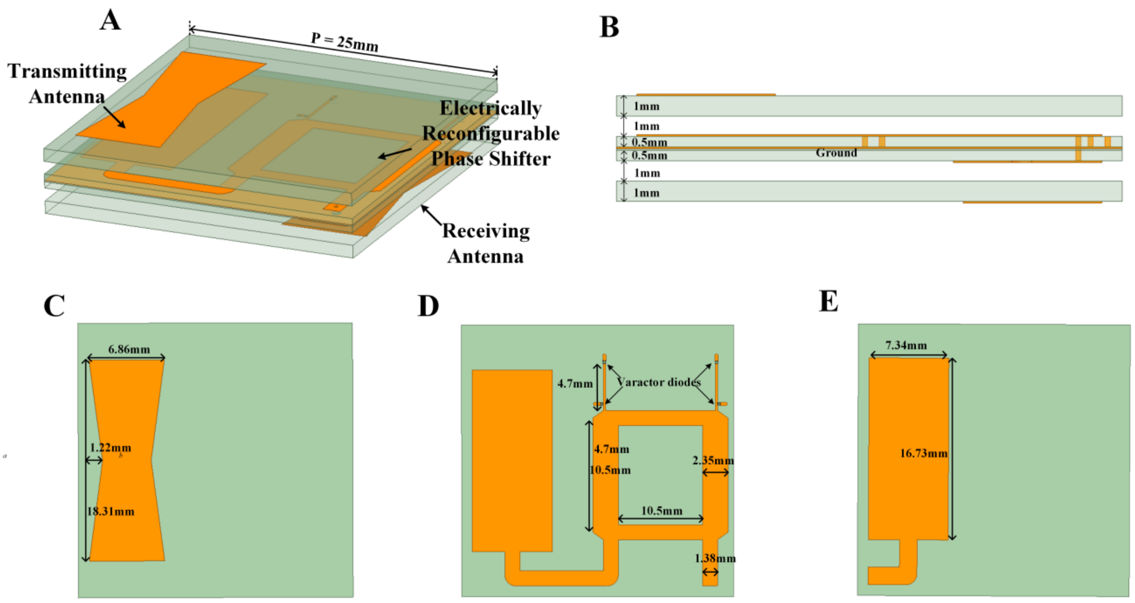

Figure S1. The geometry and the size of the meta-atom. (a) Overall structure of the reconfigurable meta-atom unit. **(b)** Cross-section view of the programmable meta-atom unit. **(c)** The top of the first layer or the bottom of the last layer structure. **(d)** The top of the middle layer structure. **(e)** The bottom of the middle layer structure.

The full-wave simulation of the structure is analyzed in the commercial EM simulation software HFSS, in which the boundary is set to be master and slave type boundary, and the excitation is set as Floquet port. In the full-wave simulation, the equivalent capacitance range of the MA46H120 varactor diode is setting from 0.2pF to 1pF with 2 ohm resistance. The simulated transmission results are presented in **Figure S2**. It can be observed that the transmission phase of the designed meta-atom can achieve 360-degree phase coverage in the working band with good linearity. Moreover, the insertion loss of the meta-atom varies from 0.9dB to 2.3dB at the frequency of 5.65GHz. Both results of the transmission amplitude and

phase can prove the meta-atom is a good candidate for two-dimensional (2D) near-field EM focusing application.

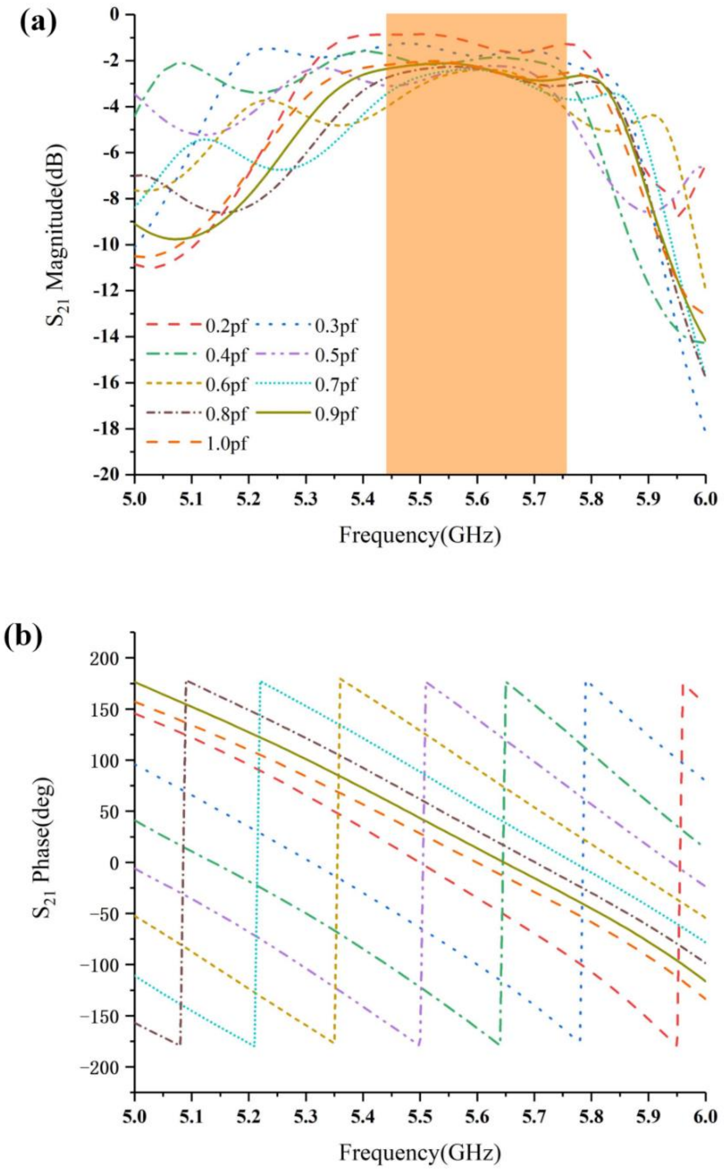

Figure S2. The unit transmission results tuning by capacitance in full-wave simulation. **(a)** Transmission magnitude results of the programmable meta-atom unit. **(b)** Transmission phase results of the programmable meta-atom unit.

S2. SVM algorithm processing flow for the gesture recognition

SVM has two commonly used operation methods for multi-classification. One is to use the one-versus-rest (OVR) mode. When judging a certain class, all other classes are regarded as

the other. This method will cause an imbalance in the number of samples of different classes, resulting in a decrease in the accuracy of classification. Therefore, the one-versus-one (OVO) mode is generally adopted, and multiple classifiers are used for voting mechanism to determine the class. Specifically, K SVMs are combined together, where N categories require $K=N*(N-1)/2$ classifiers. Each support vector machine is trained with two different types of data, and the final classifier adopts a "voting" method to determine the classification result. In this paper, because the SVM is used for classification of 18 gestures, a total of 18*17/2=153 SVM classifiers are trained.

SVM algorithm processing-flow：

Training phase：

(1) Determine the number K of SVM;

(2) Input training dataset : $\boldsymbol{X}_{\mathbf{train}}=\{\boldsymbol{x}_{\mathbf{train1}}, \boldsymbol{x}_{\mathbf{train2}}, \ldots, \boldsymbol{x}_{\mathbf{trainN1}}\}$, $\boldsymbol{Y}_{\mathbf{train}}=\{y_{\mathrm{train1}}, y_{\mathrm{train2}}, \ldots, y_{\mathrm{trainN1}}\}$, where $\boldsymbol{x}_{\mathbf{train}i} \in \boldsymbol{R}^{\mathbf{n}}$, $y_{\mathrm{train}i} \in \{0, 1, 2, ..., 17\}$, $i=1, 2, ..., \mathrm{N}_1$

(3) Use OVO mode to train 153 SVM classifiers, determine the hyperplane of each SVM at the same time, and divide the classification feature space.

Testing phase：

(1) Call the SVM model determined above;

(2) Input test dataset: $\boldsymbol{X}_{\mathbf{test}} = \{\boldsymbol{x}_{\mathbf{test1}}, \boldsymbol{x}_{\mathbf{test2}}, \ldots, \boldsymbol{x}_{\mathbf{testN2}}\}$, $\boldsymbol{Y}_{\mathbf{test}}=\{y_{\mathrm{test1}}, y_{\mathrm{test2}}, \ldots, y_{\mathrm{testN2}}\}$, where $\boldsymbol{x}_{\mathbf{test}j} \in \mathrm{R}^{\mathrm{n}}$, $y_{\mathrm{testj}} \in \{0, 1, 2, ..., 17\}$, $j=1, 2, ..., \mathrm{N}_2$

(3) Use the voting mechanism of the OVO mode to predict the category corresponding to the input feature, and finally use the input label to calculate the accuracy of the SVM classification.

S3. Linear discriminant analysis (LDA) for multiple classes

The linear discriminant analysis (LDA) is also known as Fisher's linear discriminant. First, we set the dimensionality of the input space as D and the number of recognized classes as K. Here the K is 18 and D is obviously greater than K. Next, we introduce D'>1 linear 'features' $y_k = \mathbf{w}_k^{\mathrm{T}}\mathbf{x}$, where k = 1,…,D'. These feature values can conveniently be grouped together to form a vector **y**. Similarly, the weight vectors {w_k} can be considered as the columns of a matrix **W**:

$$\mathbf{y} = \mathbf{W}^{\mathrm{T}}\mathbf{x} \tag{S1}$$

Note that any bias parameters in the definition of **y** are not included. Therefore, the generalization of the within-class covariance matrix to the case of K classes can be given as follows:

$$\mathbf{S}_{\mathbf{W}} = \sum_{k=1}^{K} \mathbf{S}_k \tag{S2}$$

where

$$\mathbf{S}_k = \sum_{n \in C_k} (\mathbf{x}_n - \mathbf{m}_k)(\mathbf{x}_n - \mathbf{m}_k)^{\mathrm{T}} \tag{S3}$$

$$\mathbf{m}_k = \frac{1}{N_k} \sum_{n \in C_k} \mathbf{x}_n \tag{S4}$$

and N_k is the number of patterns in class C_k. The generalization of the total covariance matrix can be given as follows:

$$\mathbf{S}_{\mathbf{T}} = \sum_{n \in C_k} (\mathbf{x}_n - \mathbf{m})(\mathbf{x}_n - \mathbf{m})^{\mathrm{T}} \tag{S5}$$

where m is the mean of the total data set

$$\mathbf{m} = \frac{1}{N} \sum_{n=1}^{N} \mathbf{x}_n = \frac{1}{N} \sum_{n=1}^{N} N_k \mathbf{m}_k \tag{S6}$$

and $N = \sum_k N_k$ is the total number of data points. The total covariance matrix can be decomposed into the sum of the within-class covariance matrix, given by Equation (S2), plus an additional matrix S_B, which is identified as a measure of the between-class covariance

$$\mathbf{S}_{\mathbf{T}} = \mathbf{S}_{\mathbf{W}} + \mathbf{S}_{\mathbf{B}} \tag{S7}$$

where

$$\mathbf{S}_{\mathbf{B}} = \sum_{k=1}^{K} N_k (\mathbf{m}_k - \mathbf{m})(\mathbf{m}_k - \mathbf{m})^{\mathrm{T}} \tag{S8}$$

These covariance matrices are defined in the original x-space. Now, similar matrices can be defined in the projected D'-dimensional y-space

$$s_W = \sum_{k=1}^{K} \sum_{n \in C_k} (y_n - \mu_k)(y_n - \mu_k)^T \tag{S9}$$

$$s_B = \sum_{k=1}^{K} N_k (\mu_k - \mu)(\mu_k - \mu)^T \tag{S10}$$

where

$$\mu_k = \frac{1}{N_k} \sum_{n \in C_k} y_n \tag{S11}$$

$$\mu = \frac{1}{N} \sum_{k=1}^{K} N_k \mu_k \tag{S12}$$

Finally, a scalar is constructed as follows:

$$SC_f(i) = \frac{S_B^i}{S_W^i} \tag{S13}$$

which is the Fisher score of the i-th feature. The scalar is large when the between-class covariance is large and when the within-class covariance is small.

S4. Feature extraction algorithm used for Fisher score

In order to calculate Fisher score of data set, the amplitude extreme points of the microwave data (echo coefficients) are extracted as the input features. The specific extraction algorithm is as follows:

Feature extraction algorithm processing-flow:

Input: Data set $\boldsymbol{X} = \{\boldsymbol{x}_1, \boldsymbol{x}_2, \ldots, \boldsymbol{x}_{2700}\}$, where $\boldsymbol{x}_i \in \boldsymbol{R}^n$

Process:

(1) for j=1:5 //For five spots, which means five loop times

(2) for i=1:2700 //For each sample

(3) Find the index of the extreme value (including large extreme and small extreme) in the echo coefficients data (index=1, 2, …, 201)

(4) end

(5) Similar index (for example: 35, 36, 37, 37) processing method: take the arithmetic mean

(6) Obtain the extreme value index matrix for 18 gestures under each spot (5 matrices for five spots)

(7) end

(8) According to the extreme value index matrix of Spot 1, find out the value of the corresponding index (2700*9 matrix) of the 2700 samples of Spot 1. (Same as Spot 2, Spot 3, Spot 4 and Spot 5)

(9) Join the above 5 matrices

Output: the extreme value feature matrix $\boldsymbol{Z} = \{\boldsymbol{z}_1, \boldsymbol{z}_2, \ldots, \boldsymbol{z}_{2700}\}$ of the data set $\boldsymbol{X}$;
